# Endocrine profile following stimulation with recombinant follicle stimulating hormone and luteinizing hormone versus highly purified human menopausal gonadotropin

**DOI:** 10.1186/1477-7827-12-10

**Published:** 2014-01-29

**Authors:** Antonio Requena, María Cruz, Francisco J Ruiz, Juan A García-Velasco

**Affiliations:** 1 Reproductive Medicine Department, Instituto Valenciano de Infertilidad IVI Madrid, Avenida del Talgo 68-70, Aravaca, Madrid 28023, Spain; 2 Nursing, Gynecology and Obstetrics, Pediatrics and Psychiatry Department, Faculty of Health Sciences, Rey Juan Carlos University, Avda. Atenas s/n, Alcorcón, Madrid 28922, Spain

**Keywords:** LH, FSH, Steroid profile, Follicular growth, hCG

## Abstract

**Background:**

Luteinizing hormone (LH) activity in human menopausal gonadotropin (hMG) preparations is derived from human chorionic gonadotropin (hCG) rather than LH. Therefore, we aimed to determine whether there are similarities in the endocrine and follicular profiles of serum and follicular fluid from controlled ovarian stimulation with the recombinant gonadotropins follicle-stimulating hormone plus luteinizing hormone (rFSH + rLH) or highly purified human menopausal gonadotropin (HP-hMG).

**Methods:**

We performed a prospective observational study with 50 oocyte donors that received either a combination of recombinant gonadotropins (rFSH + rLH) or a mixture of urinary gonadotropins (HP-hMG) plus purified urinary FSH (uFSH). Results were analyzed using Student’s *t*-test to compare continuous variables and the chi-squared test to compare proportions. P-values < 0.05 were considered statistically significant.

**Results:**

Although more oocytes were retrieved after treatment with recombinant than urinary gonadotropins (16.5 vs. 11.8; P = 0.049), a higher proportion of metaphase II ova (71.2% vs. 80.6%; P = 0.003) were obtained using urinary gonadotropins. On day 6 and on the day of triggering, serum steroid hormone levels were slightly but not significantly elevated in the recombinant group compared with the urinary group. In follicular fluid, no statistical differences were observed for intra-follicular levels of steroid hormones between the two protocols; ongoing pregnancy rates were similar (46.1% vs. 46.1%).

**Conclusions:**

Our data suggest that endocrinological and follicular profiles do not differ between rFSH + rLH and HP-hMG stimulation.

## Background

Within the field of controlled ovarian stimulation, in which gonadotropins are administered, the role of luteinizing hormone (LH) is widely debated. Although in the last decade a wide variety of articles have been published that compare clinical outcomes between recombinant follicle-stimulating hormone (FSH) and menotropins [1-3], there is still a need for better understanding of the differential effects of gonadotropin preparations, as well as the effect of the administration of LH activity during controlled ovarian stimulation on follicular dynamics, endocrine response, and embryo quality.

Until recently, human menopausal gonadotropin (hMG) has been the only source of exogenous LH activity, which derived from human chorionic gonadotropin (hCG) rather than from LH [4]; however, the advent of recombinant technology opened the door to the production of recombinant preparations. With almost 100% of LH content without residual FSH activity, recombinant LH (r-LH) is the first pure preparation that possesses all of the benefits of recombinant technology. Unlike hMG, r-LH provides true LH activity regarding precise and consistent LH exposure.

Because the hCG component in hMG is largely responsible for the majority of LH activity, exposure to hCG in gonadotropin stimulation may be a reliable indicator of differences in follicle growth and selection, as well as significant differences in hormonal profile [5]. It is thought that the hCG component in hMG is closely related to androgen synthesis early in ovarian stimulation to increase selective follicle growth, resulting in estrogenic mature follicles at the end of stimulation. This condition, as well as an endocrine profile leading to significantly different clinical results [6,7] implies that hMG is efficiently producing estradiol.

The question is whether the introduction of recombinant LH from the beginning of the stimulation drives an endocrine profile and follicular development similar to those obtained with exogenous urinary hCG. Thus, this study aims to compare the efficacy derived from controlled ovarian stimulation with either recombinant (rFSH + rLH) or urinary (uFSH + uLH) gonadotropins.

## Methods

We performed a prospective, parallel, observational study including 50 women from our oocyte donation program. The study was conducted at IVI Madrid throughout 2012. The sample size was feasible for a pilot study [8], and a set of patients was arbitrarily chosen to provide data that would be clinically useful. All patients provided written informed consent, and all procedures and protocols were approved by an Institutional Review Board (MAD-AR-09-2011-02) and complied with the Spanish law governing Assisted Reproductive Technologies (14/2006).

### Treatment regimen

Oocyte donors were healthy women between 18–35 years old, body mass index (BMI) 18–30 kg/m^2^, with regular menstrual cycles, no hereditary or chromosomal diseases, and normal karyotype, and who were negative when screened for sexually transmitted diseases [9]. Inclusion in the oocyte donor pool also required that the donor had at least six antral follicles at the beginning of the cycle. Donors who had polycystic ovarian syndrome (PCOS) based on Rotterdam criteria [10] or multifollicular ovaries were excluded.

Patients were assigned to each treatment group based on a quasi-experimental design comprising consecutive opportunity sampling. This means that the patients visiting each centre were consecutively included in the protocols until the investigator completed the number of patients assigned to each protocol. Treatment groups were assigned during the control visit at the start of menses, before initiating ovarian stimulation. An oral contraceptive pill (Microdiol^®^, MSD, Spain) was taken for a maximum of 21 days, starting on day 1–2 of the menses of the previous cycle. After a wash-out period of 5 days after the last pill, donors began their assigned stimulation protocol.

We performed a long GnRH agonist protocol for ovarian stimulation that consisted of down-regulation with the gonadotropin-releasing hormone (GnRH) agonist triptorelin at 0.1 mg/day (Decapeptyl^®^, Ipsen Pharma, Spain) commencing in the mid-luteal phase of the previous cycle. Once down-regulation was confirmed, subjects continued with the same dose of triptorelin (0.1 mg/day) and depending on the allocation group, received either ovarian stimulation with 150 IU r-FSH + 75 IU r-LH (Pergoveris^®^, Merck-Serono, Spain) or 75 IU HP-hMG (Menopur^®^, Ferring Pharmaceuticals, Spain) plus 75 IU u-FSH (Bravelle^®^, Ferring Pharmaceuticals, Spain). On day 6 of stimulation, each protocol was potentially followed by daily administration of 75 IU r-FSH or 75 IU u-FSH respectively, if the researcher considered this possibility after evaluating the ovarian response. A single dose of 250 μg recombinant hCG (Ovitrelle^®^, Merck-Serono, Spain) was administered as soon as three or more follicles reached a mean size of ≥17 mm. Oocyte retrieval was performed 36 h later. Follicular development was monitored by transvaginal ultrasound every 2 days until the day of hCG administration.

After oocyte retrieval, intracytoplasmic sperm injection was performed. The quality of all available embryos was evaluated, and up to two embryos were transferred on day 3 of development. Evaluated parameters on developmental day 3 included cell number, symmetry, granularity, type and percentage of fragmentation, presence of multinucleate blastomeres, and degree of compaction as previously described [11]. A top-quality embryo was described as 4–5 cells on day 2, > = 7 cells on day 3, equally sized blastomeres and < = 20% fragmentation on day 3, and no multinucleate cells.

The hormone replacement protocol for oocyte recipients has been previously described [12]. Briefly, a baseline transvaginal scan was carried out before down-regulation to ensure the uterus was normal. For all recipients who were still cycling, down-regulation was performed using an intramuscular dose of 3.75 mg triptorelin (Decapeptyl^®^; Ipsen Pharma, Spain) during the mid-luteal phase of the previous cycle. Hormone therapy was initiated on days 1–3 of the following cycle with increased doses of estradiol valerate (Progynova^®^; Schering-Plough, Spain). On day 15, an ultrasound was performed to evaluate endometrial growth. On the day after oocyte retrieval, once fertilization has been confirmed, 800 mg/day of micronized intravaginal progesterone (Progeffik; Effik Laboratories, Spain) was added to the regimen.

The β-hCG concentration was determined 13 days after embryo transfer. Implantation rate was defined as the number of intrauterine sacs compared to the number of transferred embryos and the clinical pregnancy and implantation rate was confirmed when a gestational sac with fetal heart beat was visible by ultrasound examination after 7 weeks of pregnancy.

### Sample collection

#### Serum samples

Blood samples for the analysis of circulating concentrations of endocrine parameters (estradiol, progesterone, total testosterone, and androstenedione) were assessed on day 6 of controlled ovarian stimulation and on the day of hCG administration. Serum samples were analyzed by chemiluminiscence using the Architect analyzer (Abbot Diagnostic, Spain). The analytical sensitivity of the Estradiol assay was < = 10 pg/ml, with a coefficient of variation < = 7%. The Progesterone assay demonstrated an analytical sensitivity of < = 0.1 ng/ml, with a coefficient of variation < = 7%. The analytical sensitivity and coefficient of variation derived from the Testosterone assay were 0.14 ng/ml and 8%, respectively. Finally, values obtained from the Androstenedione assay were < = 3 μg/dl for analytical sensitivity and < = 7% for coefficient of variation.

#### Follicular fluid samples

Follicular fluid was obtained during oocyte retrieval discriminating between follicles larger than and smaller than 14 mm in diameter. Follicular fluid was aspirated into 10 ml tubes using transvaginal ultrasonographic-guided oocyte retrieval. Specifically we processed, when it was possible, follicular fluid from three follicles >14 mm and from three follicles <14 mm. The needle was withdrawn and completely emptied prior to each puncture, and no culture medium was used was used in the collection tubes. After oocyte removal, follicular aspirates were centrifuged at 200 *g* for 5 min and supernatant was stored at -80°C until assayed.

### Statistical analysis

The chi-squared test and analysis of variance (ANOVA) were applied to detect statistically significant differences among the groups with regard to proportions or means. A two-sided P < 0.05 was considered statistically significant. Statistical analysis was performed using the Statistical Package for the Social Sciences, version 19.0 (IBM Corporation, New York, USA).

## Results

### Baseline

**Figure 1:**
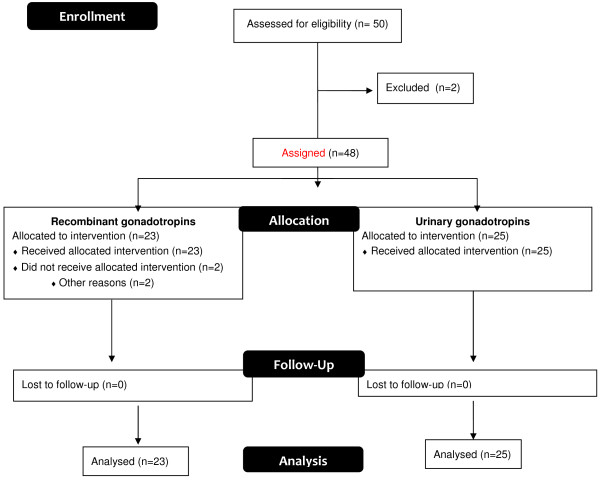
Graphical illustration of the distribution of patients.

A total of 50 oocyte donors provided informed consent and were screened for eligibility. Twenty-three donors were assigned and treated with recombinant gonadotropins (Pergoveris^®^) and 25 donors received urinary gonadotropins (Menopur^®^ + Bravelle^®^) (Figure 1).

The two treatment groups were also similar regarding demographics and baseline characteristics at the time of that stimulation was initiated (Table 1).

**Table 1:** Patient demographics and baseline serum characteristics

	**r-FSH + r-LH (n = 23)**	**HP-hMG + u-FSH (n = 25)**	**P**
*Age (years)*	24.2 +/- 2.3	26.7 +/- 1.8	0.083
*BMI (kg/m*^*2*^*)*	22.1 +/- 1.8	20.1 +/- 1.0	0.087
*Estradiol (pg/ml)*	35.5 +/- 7.3	43.7 +/- 12.3	0.212
*Progesterone (ng/ml)*	0.6 +/- 0.2	0.4 +/- 0.1	0.162
*Testosterone (ng/ml)*	0.5 +/- 0.1	0.5 +/- 0.1	0.696
*Androstenedione(ng/ml)*	1.8 +/- 0.4	2.1 +/- 0.4	0.234

### Controlled ovarian stimulation

**Figure 2:**
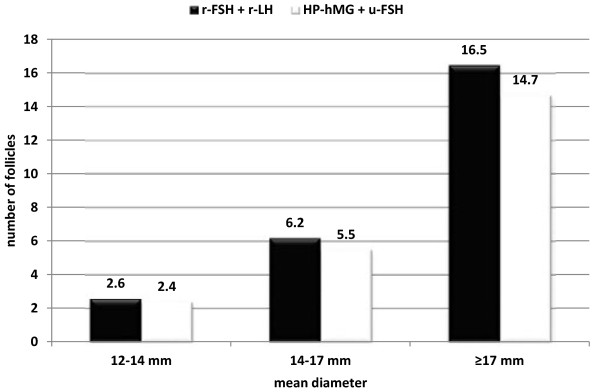
Follicular development on the day of hCG administration.

There were no significant differences in the total dose of gonadotropins (2180 +/-1350 vs. 2000 +/-1050; *P* = 0.463) or in the days of stimulation (10.7 +/- 0.9 vs. 10.5 +/- 1.0; *P* = 0.715) for recombinant and urinary gonadotropins, respectively. In addition, on the 6th day of gonadotropin administration at daily doses of 150 IU FSH and 75 IU LH, we observed no significant differences in follicular development and endocrine profile between the two stimulation protocols (Table 2); correspondingly, at the end of the stimulation no significant variations among groups were registered regarding the number of follicles sized 12–14 mm, 14–17 mm, and > = 17 mm in diameter (Figure 2), or regarding the serum estradiol, progesterone, testosterone and androstenedione concentrations (Table 3).

**Table 2:** Serum endocrine profile and follicular development on the 6th day of gonadotropin administration

	**r-FSH + r-LH (n = 23)**	**HP-hMG + u-FSH (n = 25)**	**P**
*Follicles >12 mm*	2.1 +/- 1.3	2.7 +/- 1.6	0.505
*Estradiol (pg/ml)*	436.4 +/- 181.9	323.4 +/- 80.6	0.241
*Progesterone (ng/ml)*	0.4 +/- 0.1	0.3 +/- 0.1	0.347
*Testosterone (ng/ml)*	0.6 +/- 0.2	0.5 +/- 0.1	0.683
*Androstenedione (ng/ml)*	2.9 +/- 1.4	2.0 +/- 0.5	0.248

**Table 3:** Serum endocrine profile on day of hCG administration

	**r-FSH + r-LH (n** = **23)**	**HP-hMG + u-FSH (n** = **25)**	**P**
*Estradiol (pg/ml)*	3377.7 +/-703	2547.2+/- 527.9	0.053
*Progesterone (ng/ml)*	0.9 +/- 0.2	0.7 +/- 0.2	0.156
*Testosterone (ng/ml)*	0.8 +/- 0.1	0.7 +/- 0.1	0.165
*Androstenedione (ng/ml)*	2.4 +/- 0.4	2.3 +/-0.5	0.706

Regarding the concentration of steroid hormones in follicular fluid, we registered intra-follicular levels both in follicles smaller than 14 mm and in follicles larger than 14 mm, and observed no statistical differences between the two treatments studied (Table 4).

**Table 4:** Intra-follicular levels of steroid hormones

	**Follicles < 14 mm**			**Follicles > 14 mm**		
	**r-FSH + r-LH**	**HP-hMG + u-FSH**	**P**	**r-FSH + r-LH**	**HP-hMG + u-FSH**	**P**
*Estradiol (pg/ml)*	1605350 +/- 626922	1750013 +/- 674037	0.731	1555900 +/- 353041	1733305 +/- 623936	0.580
*Progesterone (ng/ml)*	12035 +/- 4694	7904 +/- 3411	0.137	21610 +/- 4871	22174 +/- 3900	0.845
*Testosterone (ng/ml)*	9.7 +/- 3.62	9.0 +/- 3.25	0.775	6.6 +/- 1.27	7.8 +/- 2.78	0.402
*Androstenedione (ng/ml)*	12.3 +/- 5.0	15.6 +/- 6.7	0.343	9.8 +/- 1.5	11.1 +/- 4.0	0.483

### Clinical outcomes

The number of total oocytes retrieved was significantly higher with recombinant gonadotropins than with urinary gonadotropins (16.5 +/- 4.1 vs. 11.8 +/- 2.6; *P* = 0.049), while the proportion of metaphase II oocytes significantly favored the cycles stimulated with HP-hMG + uFSH (71.2% vs. 80.6%, *P* = 0.003). However, the stimulation protocols produced no significant differences in the number of metaphase II oocytes (11.8 +/- 3.7 vs. 9.5 +/- 1.8; *P* = 0.242) or the fertilization rate (67.8 +/- 12.0 vs. 78.2 +/- 10.3, P = 0.161) for recombinant and urinary gonadotropins, respectively. Treatment groups were also similar regarding the number of top-quality embryos (3.0 +/- 0.5 vs. 3.6 +/- 0.6; *P* = 0.443) and surplus frozen embryos (1.6 +/- 0.83 vs. 1.8 +/- 1.0; *P* = 0.833).

Regarding the number of transferred embryos, we observed no significant differences (P = 0.461) between recombinant gonadotropins (1.6 +/- 0.44) and urinary gonadotropins (1.8 +/- 0.36). Finally, implantation rates were 36.3% (8/22) in the recombinant group and 39.1% (9/23) in the urinary group, while the ongoing pregnancy rate was 46.1% (6/13) for both cycles.

## Discussion

The role of gonadotropins, particularly the newer and purer formulations, has been especially important to improve the efficiency of IVF. Several studies comparing the use of HP-hMG or recombinant FSH have found significant differences in both the endocrinological milieu and the follicular dynamics. Rather than resulting from the LH included in this preparation, these differences have been related to the hCG-driven LH activity added to HP-hMG. Moreover, we must also consider the differences in the type of FSH molecule, which might have been related to the variations observed between both groups. As far as we know, there have been no published studies comparing the endocrinological patterns of HP-hMG and r-FSH + r-LH.

It should be pointed out that in our study we added 75 IU of u-FSH to HP-hMG in order to use the same dose of FSH in both groups of patients, and the starting dose was also the same. We performed a long GnRH agonist protocol without reducing the initial GnRH dose at the initiation of ovarian stimulation in order to obtain a profound pituitary suppression that does not interfere in our results. Keeping these considerations in mind, we did not observe any differences regarding endocrine patterns, follicular fluid patterns, or the dynamic of follicular development.

When we analyzed our results according the number of follicles, we observed a positive trend toward stimulation with r-FSH + r-LH, although these differences did not reach statistical significance. By day 6 of stimulation, the two stimulation protocols did not differ significantly regarding the number of follicles, pointing toward similar effects on follicular growth; however, by the end of stimulation, the follicular response for the size group 12–14 mm and for larger-size follicles (14–17 mm and > = 17 mm) was advantageous for recombinant gonadotropins, which agreed with the findings of previous studies [1,2]. These numbers may be derived from the greater effectiveness of the r-FSH isoform compared with u-FSH, rather than from any effect induced by the presence of r-LH, because it is well-known that recombinant FSH leads to higher follicular recruitment [13]. However, it is also worth mentioning that the lack of significant differences might be because of the limited sample size.

Although we did not find significant differences in the total amount of gonadotropins administered and in the length of stimulation, in this study, more oocytes were also obtained with recombinant FSH, which could be considered a reflection of the greater number of follicles recruited with this FSH isoform. These data are also in agreement with previous studies [1,2,14,15]. Regarding the number of mature oocytes retrieved in each cycle, we did not observe relevant differences between the two stimulation protocols; however, when we analyzed the proportion of metaphase II cells (%MII), we found that this parameter was significantly higher in the HP-hMG group. These data also suggest that although r-FSH is more powerful, HP-hMG might favor oocyte maturation, implying that a stimulation protocol with r-FSH + r-LH would not be as effective as HP-hMG regarding oocyte maturation. This observation might be explained by the fact that hCG and r-LH differ in their half-lives and are structurally different, hence they may display different hormone-receptor interaction features [16]. Another factor that might clarify these results is the activation of determined metabolic pathways; for example, the potential of hCG to effectively convert androgens to estradiol is considered a marker of follicular health, which in turn results in better oocyte/embryo quality [5,17]. Regarding these statements, our data showed that estradiol concentrations in follicular fluid were slightly higher in the HP-hMG group, which in turn was correlated with the number of top-quality and surplus frozen embryos, which was also slightly greater with this treatment. Finally, and being conscious that our limited sample size is not suitable for evaluating clinical outcomes such as implantation and ongoing pregnancy rate, we can affirm that we did not find significant differences in either implantation or ongoing pregnancy rates; this finding is also in accordance with previous studies [18].

Our data revealed no significant differences in serum endocrine profile between the two treatments except for the estradiol concentrations on the day of hCG administration, which were higher in presence of r-FSH. However, we would like to emphasize the lack of relevant differences between these two drugs regarding progesterone levels on the day of hCG administration. According to the two-cell/two-gonadotropin theory of estrogen biosynthesis [19], theca cells under the influence of LH have the sole ability to metabolize progesterones to androgens, and these androgens are subsequently converted to estrogens through aromatization back in the granulose cells. The aromatization step is limited by the amount of precursor available, which in turn depends on LH level. It has been proposed that increased LH activity on the thecal cell in turn increases the progesterone catabolism that results in androgen formation. This hypothesis is now being questioned because it was recently reported that progesterone concentration is significantly associated with LH concentration [20]. In this later study, LH concentration was higher on the day of hCG administration when r-LH was administered; our results are in agreement with that previous data, as we obtained a higher (but not significantly so) progesterone concentration in the group that received r-LH. Our data also confirm the association between the number of developing follicles and serum progesterone concentration, suggesting that each individual follicle contributes to the collective concentration observed in the circulation. Moreover, although either the theca or the granulose cells can produce progesterone, it cannot be converted into androgens and subsequent estradiol [20], as most androgens produced by the ovary in humans go through the Δ5 pathway, which involves pregnenolone. If we consider this new point of view and the absence of statistically significant differences, we might suggest that r-LH activates this Δ5 steroid metabolic pathway as effectively as the hCG contained in HP-hMG. Regarding intrafollicular assessments, we did not observe statistical differences for follicles larger or smaller than 14 mm; this latter result confirms the absence of significant differences noted in serum observations.

## Conclusions

In summary, serum and follicular fluid hormonal profiles were similar when r-FSH + r-LH was compared with HP-hMG. This finding leads us to conclude that the metabolic activity of r-LH is comparable to that of hCG.

## Abbreviations

ANOVA: Analysis of variance; E2: Estradiol; FSH: Follicle stimulating hormone; GnRH: Gonadotropin-releasing hormone; hCG: Human chorionic gonadotropin; hMG: Human menopausal gonadotropin; HP-hMG: Highly purified human menopausal gonadotropin; IVF: In vitro fertilization; LH: Luteinizing hormone; MII: Metaphase II; P4: Progesterone; PCOS: Polycystic ovarian syndrome; T: Testosterone.

## Competing interests

The authors declare that they have no competing interests.

## Authors’ contributions

AR played a role in conception and design, analysis and interpretation of the data, drafting the manuscript and revising it critically for important intellectual content, and final approval of the manuscript. MC was involved in drafting the manuscript. FR took part in data collection and interpretation. JG-V was involved in drafting the manuscript, providing important intellectual content and providing final approval. All authors read and approved the final manuscript.
